# Neoadjuvant chemotherapy followed by surgery versus concurrent chemoradiotherapy in patients with stage IIB cervical squamous cell carcinoma: a retrospective cohort study

**DOI:** 10.1186/s12885-024-12411-6

**Published:** 2024-05-29

**Authors:** Xin-Bin Pan, Yan Lu, You-Sheng Wei, De-Sheng Yao

**Affiliations:** 1 Department of Radiation Oncology, Guangxi Medical University Cancer Hospital, Nanning, Guangxi 530021 P.R. China; 2 Department of Gynecologic Oncology, Guangxi Medical University Cancer Hospital, No. 71 Hedi Road, Qingxiu District, Nanning, Guangxi 530021 P.R. China

**Keywords:** Cervical squamous cell carcinoma, Stage IIB, Surgery, Neoadjuvant chemotherapy, Concurrent chemoradiotherapy, Survival

## Abstract

**Purpose:**

This study aims to compare treatment outcomes between neoadjuvant chemotherapy (NACT) followed by surgery and concurrent chemoradiotherapy (CCRT) in patients with stage IIB cervical squamous cell carcinoma (CSCC).

**Materials and methods:**

We conducted a retrospective cohort study involving patients with stage IIB CSCC treated at Guangxi Medical University Cancer Hospital between June 2012 and June 2019. We compared overall survival (OS), locoregional-free survival (LRFS), and distant metastasis-free survival (DMFS) between the NACT + surgery and CCRT groups.

**Results:**

A total of 257 patients were enrolled: 165 underwent NACT + surgery and 92 received CCRT. Before propensity score matching, the NACT + surgery group exhibited lower 5-year OS (68.2% vs. 85.6%; hazard ratio [HR] = 2.50, 95% confidence interval [CI]: 1.26–4.96; *P* = 0.009), LRFS (85.2% vs. 96.9%; HR = 5.88, 95% CI: 1.33–25.94; *P* = 0.019), and DMFS (81.9% vs. 97.4%; HR = 6.65, 95% CI: 1.51–29.23; *P* = 0.012) compared to the CCRT group. After propensity score matching, OS, LRFS, and DMFS remained worse in the NACT + surgery group compared to the CCRT group.

**Conclusion:**

NACT followed by surgery is associated with decreased OS, LRFS, and DMFS compared to CCRT among patients with stage IIB CSCC.

## Introduction

Cervical squamous cell carcinoma (CSCC) presents a significant health challenge globally, especially affecting women in developing countries [1]. In these regions, patients frequently present with advanced stages of the disease [2]. While concurrent chemoradiotherapy (CCRT) has been established as the standard treatment modality for these locally advanced CSCC [3, 4], surgical intervention is also proposed as a viable alternative [5, 6]. However, the efficacy of surgery in comparison to CCRT is currently a subject of debate. Divergent studies have reported variable outcomes. Some studies indicate that surgery may yield overall survival (OS) rates comparable to those achieved with CCRT [7–10]. In contrast, others indicate worse survival outcomes with surgical approaches [11, 12]. Our study is designed to compare the survival outcomes between neoadjuvant chemotherapy (NACT) followed by surgery and CCRT in patients with stage IIB CSCC.

## Materials and methods

### Patients

This retrospective cohort study analyzed CSCC patients treated at Guangxi Medical University Cancer Hospital from June 2012 to June 2019. Inclusion criteria were as follows: [1] pathologically confirmed cervical cancer, [2] squamous cell carcinoma, [3] stage IIB according to the International Federation of Gynaecology and Obstetrics (FIGO) staging system [13], [4] patients received CCRT or NACT + surgery. Exclusion criteria were as follows: [1] patients did not receive any treatments, [2] patients had incomplete data, [3] patients did not complete treatments, [4] patients underwent surgery alone, [5] patients received CCRT combined with neoadjuvant or adjuvant therapies.

Clinical variables collected included Eastern Cooperative Oncology Group (ECOG) status, age, tumor grade, human papillomavirus (HPV) infection status, hemoglobin levels, and tumor diameter. Tumor status, lymph node status, and systemic status were recorded according to clinical, imaging, pathological findings [13]. 

### Surgery

Surgical procedures comprised laparoscopic or open hysterectomy, as detailed in our previous studies [14–16]. Procedures included Piver-Rutledge class III abdominal hysterectomy, bilateral pelvic lymphadenectomy, and lower para-aortic lymph node sampling, all performed by expert gynecologic oncologists. Before surgery, 1 mL of carbon nanoparticles was injected into the cervix surrounding the tumor. The injection process lasted at least 3 min. The first lymph nodes to exhibit black staining after the injection initiation were identified as the sentinel lymph nodes. The number and location of these sentinel lymph nodes were recorded. Subsequently, the sentinel lymph nodes were excised and sent for pathological examination.

Prior to surgery, patients underwent 1 to 3 cycles of platinum-based NACT every three weeks. The surgery was scheduled 3–4 weeks after the final NACT cycle, based on the clinical response. Post-surgery, patients identified with risk prognostic factors were administered tailored adjuvant therapies. These therapies included options like chemotherapy, radiotherapy, or concurrent chemoradiotherapy [17, 18]. The prognostic risk factors guiding these decisions included more than one third stromal invasion, capillary lymphatic space involvement, a tumor diameter exceeding 4 cm, positive pelvic lymph nodes, positive surgical margins, and microscopic involvement of the parametrium.

### Concurrent chemoradiotherapy

Radiotherapy combined pelvic external beam radiotherapy (48–50 Gy over 24–25 fractions using intensity-modulated radiotherapy) with high-dose-rate intracavitary brachytherapy (28–35 Gy over 4–5 fractions targeting the high-risk clinical target volume). Cone beam computed tomography was used for daily verification during the first week of treatment, followed by weekly verification thereafter.

The radiation doses were carefully adjusted to minimize impact on surrounding structures like the tumor, rectum, and bladder. To standardize bladder filling and reduce interaction motion, we employed a systematic drinking protocol. Patients were instructed to drink 500 mL of water 45 min before each radiotherapy session. This protocol was consistently followed to ensure reproducible bladder volumes, which is critical for maintaining the precision of radiation delivery and minimizing dose variations to surrounding organs at risk.

Concurrent chemotherapy involved weekly intravenous cisplatin (30–40 mg/m2/d1) or nedaplatin (50 mg/m2/d1) administered during the external beam radiotherapy period.

### Endpoints

The primary endpoint of our study was OS, defined as the duration from diagnosis to death from any cause. Secondary endpoints were locoregional-free survival (LRFS) and distant metastasis-free survival (DMFS), which measured the time from diagnosis to either locoregional recurrence or distant metastasis, respectively.

### Statistical analysis

Patients were divided into two groups: those receiving NACT + surgery, and those undergoing CCRT. We categorized continuous variables like age and hemoglobin levels at their median values. Tumor diameter, a continuous variable, was grouped at 4 cm [19]. Categorical variables, including ECOG status, age, tumor grade, HPV infection status, hemoglobin levels, and tumor diameter, were analyzed using the χ^2^ test or Fisher’s exact test.

For survival analysis, we employed the Kaplan-Meier method, using log-rank tests to compare OS, LRFS, and DMFS between NACT + surgery and CCRT groups. Multivariable proportional hazards models, adjusted for ECOG status, age, tumor grade, HPV infection status, hemoglobin levels, tumor diameter, and treatment modalities, were used to identify independent prognostic factors. The results were presented as hazard ratios (HRs) with 95% confidence intervals (CIs).

To minimize selection bias between the NACT + surgery and CCRT groups, we used a matched case-control approach through propensity score matching (PSM). The scores were calculated using a logistic regression model with CCRT as the dependent variable. We matched cases one-to-one without replacement, based on nearest-neighbor matching on the logit of the propensity score, considering confounding factors and a caliper of 0.01.

All statistical analyses were performed using SPSS Statistics Version 26.0 (IBM Co., Armonk, NY, USA) and R software (version 4.2.2). A two-tailed P value below 0.05 was considered statistically significant.

Ethical approval for this study was obtained from the Guangxi Medical University Cancer Hospital Ethics Committee. The study was conducted in compliance with the principles outlined in the Declaration of Helsinki. However, informed consent was not obtained due to the retrospective nature of the study.

## Results

### Patient characteristics

**Fig. 1:**
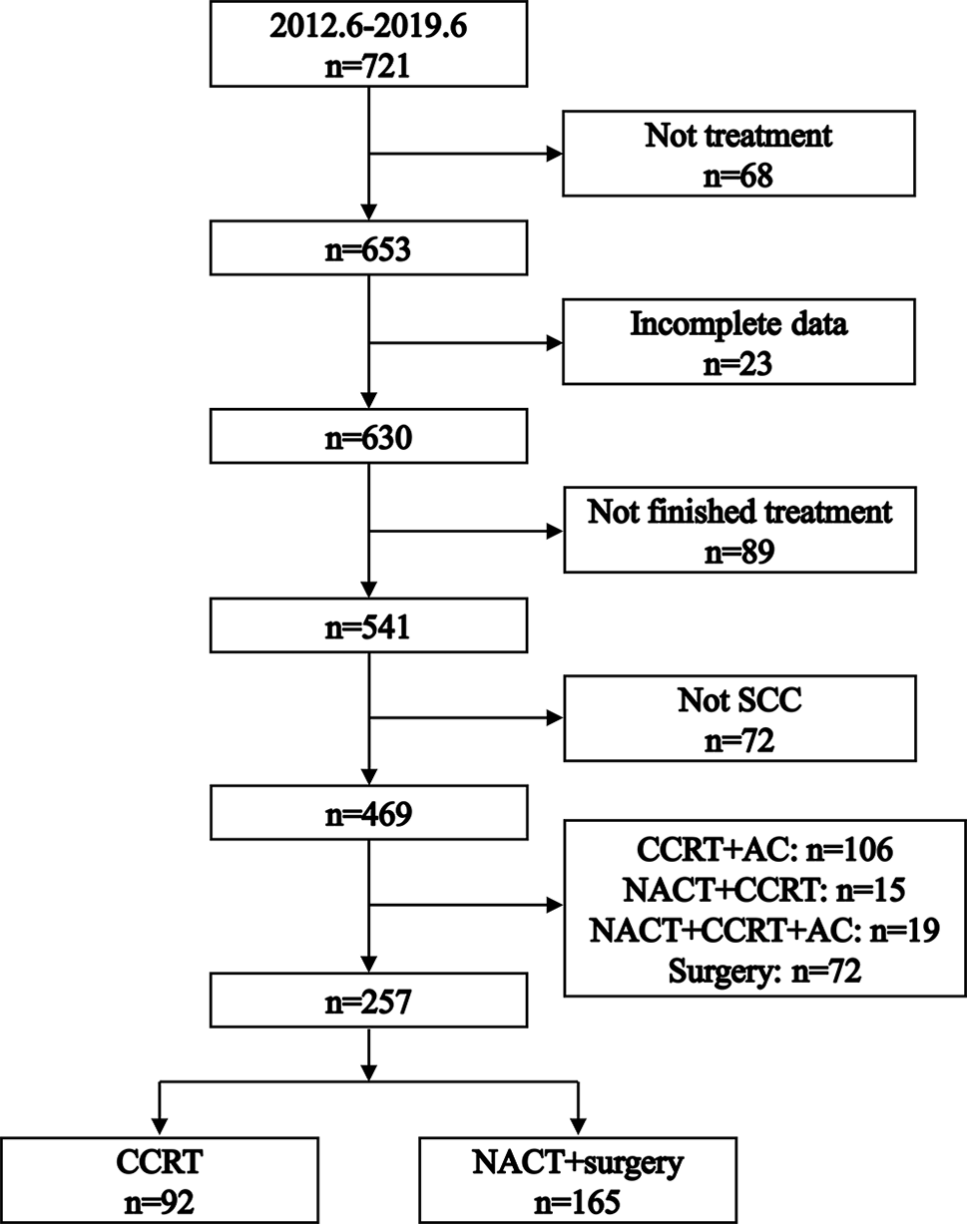
Flowchart illustrating the process of patient selection. SCC: squamous cell carcinoma. NACT: neoadjuvant chemotherapy. CCRT: concurrent chemoradiotherapy. AC: adjuvant chemotherapy

The selection process is illustrated in Fig. 1. Our study included 257 patients: 165 (64.2%) underwent NACT + surgery and 92 (35.8%) received CCRT. Patient characteristics, both before and after PSM, are detailed in Table 1. Prior to PSM, baseline characteristics like age, ECOG status, and HPV infection status showed imbalances between the NACT + surgery and CCRT groups. After PSM, 69 patients who received NACT + surgery and 69 patients who received CCRT were matched. Patient characteristics showing no significant differences across all covariates (*P* > 0.05).

**Table 1:** Patient characteristics

	Unmatched cohort		*P*	PSM cohort		*P*
	CCRT (*n* = 92)	NACT + surgery (*n* = 165)		CCRT (*n* = 69)	NACT + surgery (*n* = 69)	
Age (year)			< 0.001			0.999
≤ 54	32 (34.8%)	103 (62.4%)		31 (44.9%)	31 (44.9%)	
> 54	60 (65.2%)	62 (37.6%)		38 (55.1%)	38 (55.1%)	
ECOG			0.008			0.999
0	19 (20. 7%)	62 (37.6%)		18 (26.1%)	18 (26.1%)	
1	73 (79.3%)	103 (62.4%)		51 (73.9%)	51 (73.9%)	
Grade			0.901			0.999
III	41 (44.6%)	75 (45.5%)		31 (44.9%)	32 (46.4%)	
II	24 (26.1%)	48 (29.1%)		16 (23.2%)	16 (23.2%)	
I	1 (1.1%)	2 (1.2%)		0 (0.0%)	0 (0.0%)	
unknown	26 (28.2%)	40 (24.2%)		22 (31.9%)	21 (30.4%)	
Hgb (g/L)			0.860			0.999
≤ 118	45 (48.9%)	84 (50.9%)		36 (52.2%)	37 (53.6%)	
> 118	47 (51.1%)	81 (49.1%)		33 (47.8%)	32 (46.4%)	
HPV			0.014			0.941
negative	9 (9.8%)	14 (8.5%)		5 (7.2%)	4 (5.8%)	
positive	65 (70.7%)	90 (54.5%)		48 (69.6%)	49 (71.0%)	
unknown	18 (19.5%)	61 (37.0%)		16 (23.2%)	16 (23.2%)	
Diameter (cm)			0.100			0.999
≤ 4	38 (41.3%)	50 (30.3%)		27 (39.1%)	27 (39.1%)	
>4	54 (58.7%)	115 (69.7%)		42 (60.9%)	42 (60.9%)	

ECOG: Eastern Cooperative Oncology Group. Hgb: hemoglobin. HPV: human papilloma virus. PSM: propensity score matching. CCRT: concurrent chemoradiotherapy. NACT: neoadjuvant chemotherapy

Within the CCRT group, the median number of concurrent chemotherapy cycles was 4 (interquartile range: 3–5 cycles). In the NACT + surgery group, 76 (46.1%) patients receive NACT + surgery alone, 51 (30.9%) patents receive radiotherapy after NACT + surgery, 5 (3.0%) patients received chemotherapy after NACT + surgery, and 33 (20.0%) patients received CCRT after NACT + surgery. Post-surgery, 68 (41.2%) patients in the NACT + surgery group were diagnosed with lymph node metastases.

### Logistic regression for factors associated with NACT + surgery

**Fig. 2:**
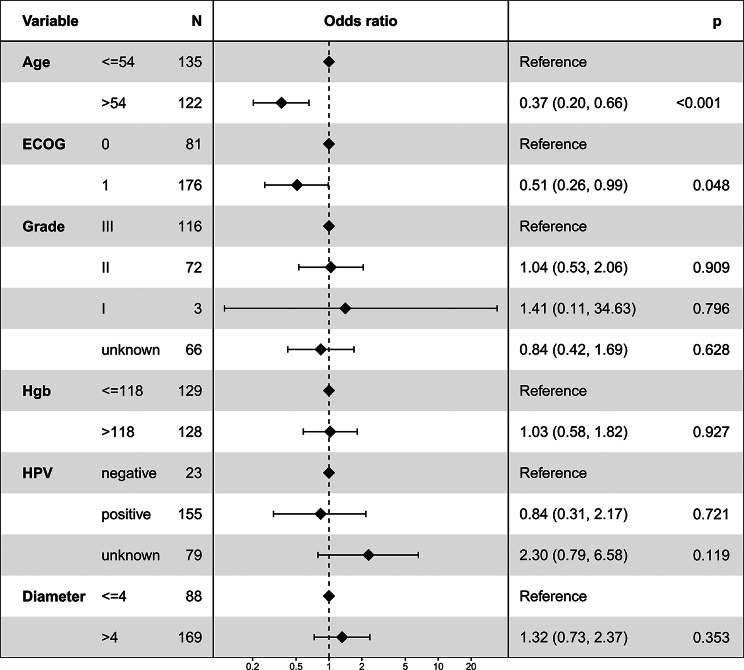
Logistic regression analysis depicting factors associated with the utilization of neoadjuvant chemotherapy followed by surgery

Figure 2 presents the logistic regression analysis results, exploring factors influencing the choice of NACT + surgery. The analysis revealed a significant association between the selection of NACT + surgery and both age and ECOG status. Specifically, patients older than 54 years were less likely to undergo NACT + surgery (odds ratio = 0.37, 95% CI: 0.20–0.66; *P* < 0.001). Similarly, a lower likelihood of opting for NACT + surgery was observed in patients with an ECOG score of 1 (odds ratio = 0.51, 95% CI: 0.26–0.99; *P* = 0.048).

### Overall survival

**Fig. 3:**
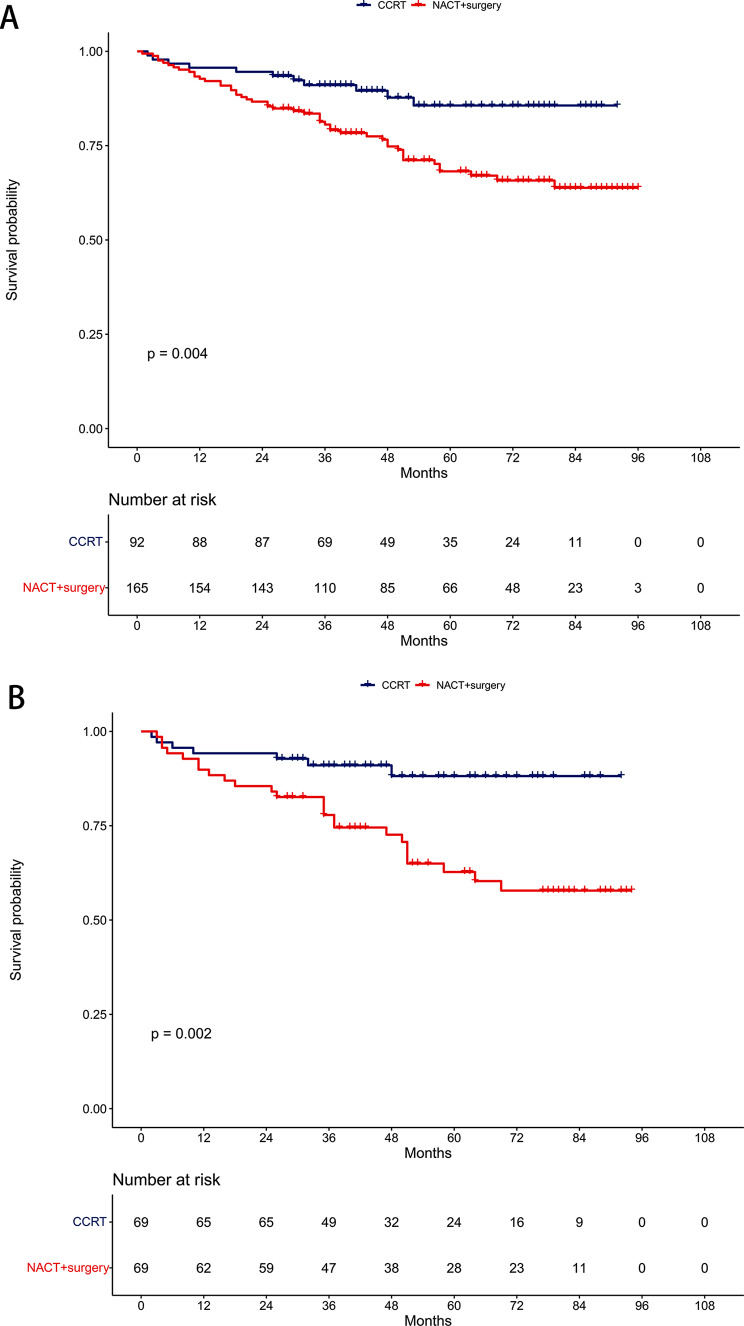
Comparison of overall survival between the neoadjuvant chemotherapy followed by surgery and concurrent chemoradiotherapy groups. (**A**) Unmatched cohort. (**B**) Propensity-matched cohort

Before PSM, the 5-year OS rates were 68.2% in the NACT + surgery group and 85.6% in the CCRT group (*P* = 0.004, Fig. 3A). Multivariable proportional hazards models revealed that NACT + surgery was an independent risk prognostic factor for OS (HR = 2.50, 95% CI: 1.26–4.96; *P* = 0.009, Table 2).

**Table 2:** Multivariable proportional hazards regressions analyzing survivals between the neoadjuvant chemotherapy followed by surgery and concurrent chemoradiotherapy groups before propensity score matching

	OS			LRFS			DMFS		
	HR	95% CI	*P*	HR	95% CI	*P*	HR	95% CI	*P*
Age (year)									
≤54	reference			reference			reference		
>54	0.83	0.47–1.44	0.500	1.12	0.44–2.86	0.812	1.06	0.45–2.48	0.896
ECOG									
0	reference			reference			reference		
1	1.21	0.68–2.13	0.514	0.89	0.36–2.22	0.809	0.92	0.29–2.18	0.798
Grade									
III	reference			reference			reference		
II	0.85	0.44–1.62	0.611	0.47	0.15–1.48	0.197	1.13	0.45–2.81	0.798
I	/	/	/	/	/	/	4.90	0.60-39.77	0.137
unknown	0.95	0.49–1.83	0.877	0.65	0.22–1.93	0.435	0.82	0.27–2.42	0.712
Hgb (g/L)									
≤118	reference			reference			reference		
>118	0.91	0.53–1.55	0.729	0.58	0.23–1.48	0.253	1.31	0.58–2.98	0.517
HPV									
negative	reference			reference			reference		
positive	0.73	0.31–1.69	0.458	2.38	0.31–18.49	0.408	0.60	0.16–2.20	0.439
unknown	0.75	0.31–1.83	0.529	1.50	0.17–12.97	0.711	0.93	0.26–3.40	0.915
Diameter (cm)									
≤4	reference			reference			reference		
>4	0.89	0.50–1.59	0.698	1.65	0.54–5.06	0.382	0.97	0.40–2.35	0.950
Treatment									
CCRT	reference			reference			reference		
NACT + surgery	2.50	1.26–4.96	0.009	5.88	1.33–25.94	0.019	6.65	1.51–29.23	0.012

ECOG: Eastern Cooperative Oncology Group. Hgb: hemoglobin. HPV: human papilloma virus. HR: hazard ratio. CI: confidence interval. OS: overall survival. LRFS: locoregional-free survival. DMFS: distant metastasis-free survival. CCRT: concurrent chemoradiotherapy. NACT: neoadjuvant chemotherapy

After PSM, the 5-year OS rates were 62.7% in the NACT + surgery group and 88.2% in the CCRT group (*P* = 0.002, Fig. 3B). Similarly, NACT + surgery remained an independent risk prognostic factor for OS in multivariable proportional hazards models (HR = 3.67, 95% CI: 1.58–8.52; *P* = 0.003, Table 3).

**Table 3:** Multivariable proportional hazards regressions evaluating survivals between the neoadjuvant chemotherapy followed by surgery and concurrent chemoradiotherapy groups after propensity score matching

	OS			LRFS			DMFS		
	HR	95% CI	*P*	HR	95% CI	*P*	HR	95% CI	*P*
Age (year)									
≤54	reference			reference			reference		
>54	0.51	0.23–1.13	0.096	1.51	0.37–6.12	0.565	0.48	0.11–2.07	0.325
ECOG									
0	reference			reference			reference		
1	1.67	0.66–4.22	0.280	1.10	0.25–4.87	0.902	1.79	0.29–11.12	0.531
Grade									
III	reference			reference			reference		
II	0.41	0.15–1.52	0.091	0.26	0.03–2.39	0.234	0.77	0.17–3.54	0.741
I	/	/	/	/	/	/	/	/	/
unknown	0.61	0.25–1.45	0.261	0.56	0.12–2.54	0.451	0.29	0.05–1.80	0.183
Hgb (g/L)									
≤118	reference			reference			reference		
>118	1.11	0.51–2.44	0.787	0.52	0.10–2.67	0.437	2.28	0.52–9.90	0.272
HPV									
negative	reference			reference			reference		
positive	1.14	0.24–5.40	0.873	/	/	/	/	/	/
unknown	0.94	0.18–4.81	0.934	/	/	/	/	/	/
Diameter (cm)									
≤4	reference			reference			reference		
>4	0.76	0.34–1.70	0.503	1.33	0.26–6.81	0.731	0.50	0.12–2.10	0.346
Treatment									
CCRT	reference			reference			reference		
NACT + surgery	3.67	1.58–8.52	0.003	8.31	1.03–67.32	0.047	10.94	1.34–89.21	0.026

ECOG: Eastern Cooperative Oncology Group. Hgb: hemoglobin. HPV: human papilloma virus. HR: hazard ratio. CI: confidence interval. OS: overall survival. LRFS: locoregional-free survival. DMFS: distant metastasis-free survival. CCRT: concurrent chemoradiotherapy. NACT: neoadjuvant chemotherapy

### Locoregional-free survival

**Fig. 4:**
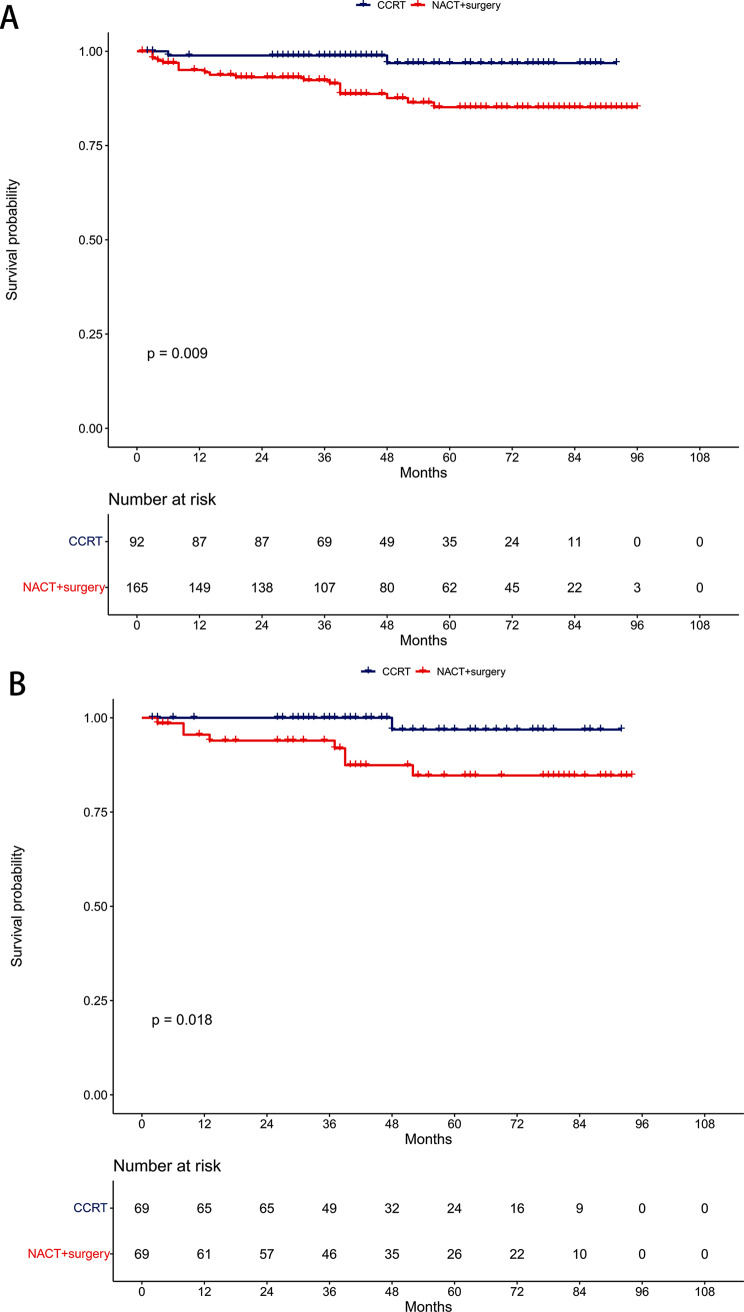
Comparison of locoregional-free survival between the neoadjuvant chemotherapy followed by surgery and concurrent chemoradiotherapy groups. (**A**) Unmatched cohort. (**B**) Propensity-matched cohort

Pre-PSM, the 5-year LRFS rates were 85.2% in the NACT + surgery group and 96.9% in the CCRT group (*P* = 0.009, Fig. 4A). Multivariable proportional hazards models demonstrated that NACT + surgery was an independent risk prognostic factor for LRFS (HR = 5.88, 95% CI: 1.33–25.94; *P* = 0.019, Table 2).

Post-PSM, the 5-year LRFS rates were 84.7% in the NACT + surgery group and 96.9% in the CCRT group (*P* = 0.018, Fig. 4B). NACT + surgery retained its significance as an independent risk prognostic factor for LRFS in multivariable proportional hazards models (HR = 8.31, 95% CI: 1.03–67.32; *P* = 0.047, Table 3).

### Distant metastasis-free survival

**Fig. 5:**
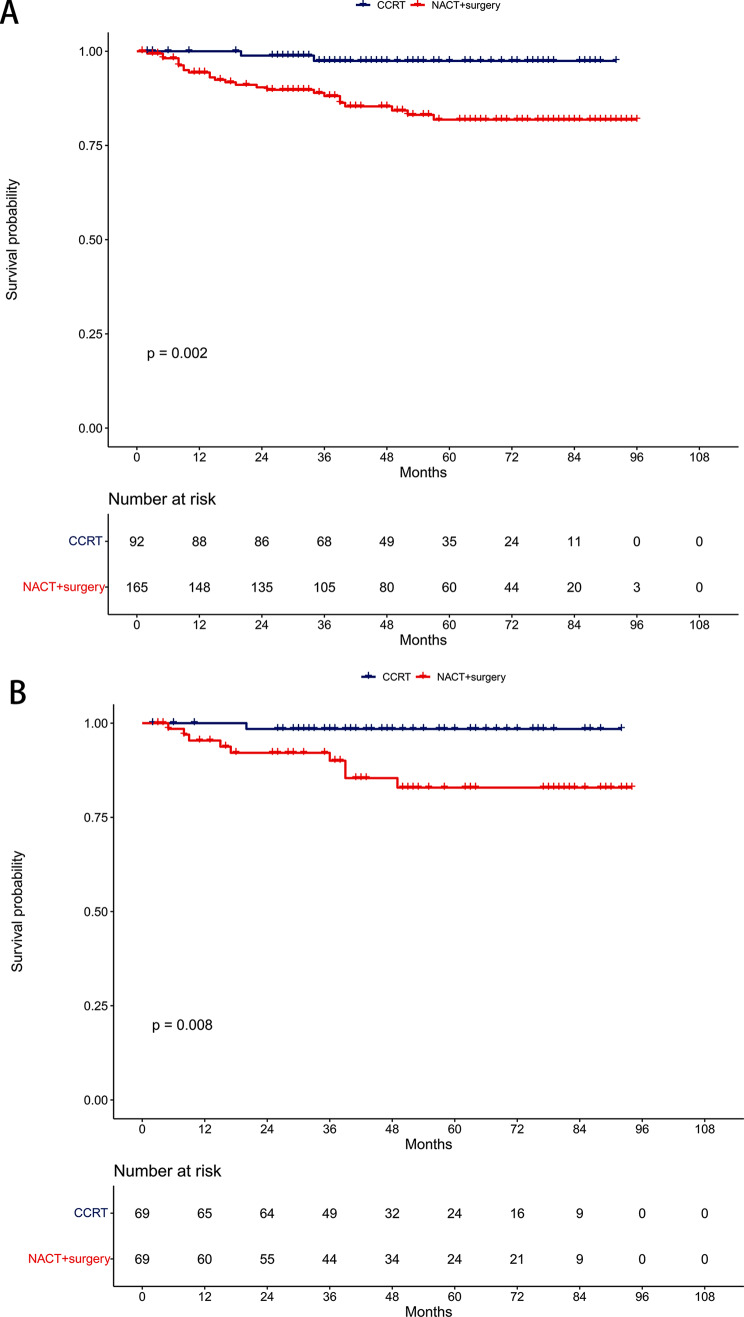
Comparison of distant metastasis-free survival between the neoadjuvant chemotherapy followed by surgery and concurrent chemoradiotherapy groups. (**A**) Unmatched cohort. (**B**) Propensity-matched cohort

Before PSM, the 5-year DMFS rates were 81.9% in the NACT + surgery group and 97.4% in the CCRT group (*P* = 0.002, Fig. 5A). Multivariable proportional hazards models revealed NACT + surgery as an independent risk prognostic factor for DMFS (HR = 6.65, 95% CI: 1.51–29.23; *P* = 0.012, Table 2).

After PSM, the 5-year DMFS rates were 82.9% in the NACT + surgery group and 98.5% in the CCRT group (*P* = 0.008, Fig. 5B). NACT + surgery continued to be an independent risk prognostic factor for DMFS in multivariable proportional hazards models (HR = 10.94, 95% CI: 1.34–89.21; *P* = 0.026, Table 3).

## Discussion

Our study offered a pivotal understanding that NACT before surgery did not improve survival outcomes in stage IIB CSCC patients. This aligns with the existing perspective that stage IIB CSCC is an unresectable disease [20]. Consequently, CCRT remains the fundamental treatment strategy. These results strongly suggested that surgery as an initial radical approach should not be considered for this patient subgroup [21]. 

Our results may suggest that the delay in surgery due to NACT might compromise survival rates. The initial decrease in 5-year LRFS and DMFS associated with NACT + surgery could adversely affect OS, indicating that NACT-induced tumor size reduction may not be adequate for effective radical surgery in this specific patient group [22]. 

Despite being the preferred treatment, CCRT for stage IIB CSCC may lead to significant long-term radiation-induced complications, including ovarian failure, vaginal fibrosis, enteritis, fistulas, bowel obstruction, and lymphedema [23]. These adverse effects significantly impact the quality of life for patients during long-term survival [24]. This reality might incline clinicians toward considering surgical interventions for these patients. However, recent studies indicate that achieving an optimal dose distribution, compliant with the treatment planning objectives of the EMBRACE II protocol, is more feasible when the external beam radiotherapy dose is limited to 45 Gy [25]. Moreover, the systematic use of an interstitial brachytherapy component can increase the dose delivered to 98% of the volume from 83 ± 14 Gy to 92 ± 13 Gy without increasing the dose to organs at risk. Adhering to these updated international guidelines could substantially reduce the complications associated with CCRT and increase the locoregional control rates [13]. 

However, the approach to treating stage IIB CSCC is not always consistent in clinical practice, primarily due to the subjective nature of staging, which relies on physical examination. This variability often leads to different surgical decisions among clinicians [26, 27]. In certain cases, experts might recommend surgery, basing their decision on specific factors such as tumor size, histopathological grade, and lymph node status [28]. 

Moreover, recent advancements in chemotherapy regimens, particularly those involving platinum and cisplatin, have shown notable effectiveness in CSCC [29]. Platinum-based NACT has been found to reduce the primary tumor burden, which could lead to a higher rate of complete resection. Additionally, Platinum-based NACT is believed to have the capability to prevent cancer cell implantation and to eradicate circulating cancer cells, thereby potentially diminishing subclinical metastasis. This could lead to improved DMFS, a hypothesis that is gaining support from various studies [30–32]. 

The primary aim of using NACT is to enhance treatment outcomes compared to CCRT. However, findings from a single-center, phase III, randomized controlled trial presented a different picture [12]. This study reported a 5-year disease-free survival of 69.3% in the NACT + surgery group, compared to 76.7% in the CCRT group (HR = 1.38, 95% CI: 1.02–1.87; *P* = 0.038). Interestingly, the corresponding 5-year OS rates were quite similar: 75.4% for NACT + surgery and 74.7% for CCRT (HR = 1.025, 95% CI: 0.752–1.398; *P* = 0.87). This suggested that NACT + surgery did not significantly improve survival rates over CCRT.

A notable observation from this trial was that the reduced disease-free survival in the NACT + surgery group did not lead to a worse OS. This could be attributed to the effectiveness of salvage treatments following recurrence. It is important to note that a majority of first recurrences (102 out of 162 recurrences, constituting 62.96%) were localized, emphasizing the critical role of local control. After recurrence, 30.0% of patients in the NACT + surgery group received local radiotherapy, compared to just 11.0% in the CCRT group. This finding underscored the importance of local control in managing recurrent CSCC and illustrated the complexity inherent in treatment decisions and their long-term implications.

A major strength of our study was the consistent quality of treatment delivery by a multidisciplinary team of experts. However, its retrospective nature introduced potential limitations, including possible confounders between the NACT + surgery and CCRT groups. Although we employed PSM and multivariable proportional hazards models to address these biases, our findings need to be validated by larger-scale studies conducted at diverse centers.

In conclusion, our research highlights that NACT + surgery decreased OS, LRFS, and DMFS compared to CCRT in treating stage IIB CSCC, emphasizing the need for cautious consideration in treatment planning.

## Data Availability

The data are available from the corresponding author upon request.

## Abbreviations

CSCC: Cervical squamous cell carcinoma
CCRT: Concurrent chemoradiotherapy
OS: Overall survival
NACT: Neoadjuvant chemotherapy
FIGO: The International Federation of Gynaecology and Obstetrics
ECOG: Eastern Cooperative Oncology Group
HPV: Human papilloma virus
LRFS: Locoregional-free survival
DMFS: Distant metastasis-free survival
PSM: Propensity score matching
HR: Hazard ratio
CI: Confidence interval
